# Comparison of ultrahigh and standard resolution photon-counting CT angiography of the femoral arteries in a continuously perfused *in vitro* model

**DOI:** 10.1186/s41747-023-00398-x

**Published:** 2023-12-18

**Authors:** Philipp Gruschwitz, Viktor Hartung, Süleyman Ergün, Dominik Peter, Sven Lichthardt, Henner Huflage, Robin Hendel, Pauline Pannenbecker, Anne Marie Augustin, Andreas Steven Kunz, Philipp Feldle, Thorsten Alexander Bley, Jan-Peter Grunz

**Affiliations:** 1 Department of Diagnostic and Interventional Radiology, University Hospital of Würzburg, Würzburg, Germany; 2 Institute of Anatomy and Cell Biology, University of Würzburg, Würzburg, Germany; 3 Department of General, Visceral, Transplant, Vascular, and Pediatric Surgery, University Hospital of Würzburg, Würzburg, Germany

**Keywords:** CT angiography, Femoral arteries, Photon-counting computed tomography (CT), Small pixel effect, Ultrahigh resolution

## Abstract

**Background:**

With the emergence of photon-counting CT, ultrahigh-resolution (UHR) imaging can be performed without dose penalty. This study aims to directly compare the image quality of UHR and standard resolution (SR) scan mode in femoral artery angiographies.

**Methods:**

After establishing continuous extracorporeal perfusion in four fresh-frozen cadaveric specimens, photon-counting CT angiographies were performed with a radiation dose of 5 mGy and tube voltage of 120 kV in both SR and UHR mode. Images were reconstructed with dedicated convolution kernels (soft: Body-vascular (Bv)48; sharp: Bv60; ultrasharp: Bv76). Six radiologists evaluated the image quality by means of a pairwise forced-choice comparison tool. Kendall’s concordance coefficient (*W*) was calculated to quantify interrater agreement. Image quality was further assessed by measuring intraluminal attenuation and image noise as well as by calculating signal-to-noise ratio (SNR) and contrast-to-noise ratios (CNR).

**Results:**

UHR yielded lower noise than SR for identical reconstructions with kernels ≥ Bv60 (*p* < 0.001). UHR scans exhibited lower intraluminal attenuation compared to SR (Bv60: 406.4 ± 25.1 *versus* 418.1 ± 30.1 HU; *p* < 0.001). Irrespective of scan mode, SNR and CNR decreased while noise increased with sharper kernels but UHR scans were objectively superior to SR nonetheless (Bv60: SNR 25.9 ± 6.4 *versus* 20.9 ± 5.3; CNR 22.7 ± 5.8 *versus* 18.4 ± 4.8; *p* < 0.001). Notably, UHR scans were preferred in subjective assessment when images were reconstructed with the ultrasharp Bv76 kernel, whereas SR was rated superior for Bv60. Interrater agreement was high (*W* = 0.935).

**Conclusions:**

Combinations of UHR scan mode and ultrasharp convolution kernel are able to exploit the full image quality potential in photon-counting CT angiography of the femoral arteries.

**Relevance statement:**

The UHR scan mode offers improved image quality and may increase diagnostic accuracy in CT angiography of the peripheral arterial runoff when optimized reconstruction parameters are chosen.

**Key points:**

• UHR photon-counting CT improves image quality in combination with ultrasharp convolution kernels.

• UHR datasets display lower image noise compared with identically reconstructed standard resolution scans.

• Scans in UHR mode show decreased intraluminal attenuation compared with standard resolution imaging.

**Graphical Abstract:**

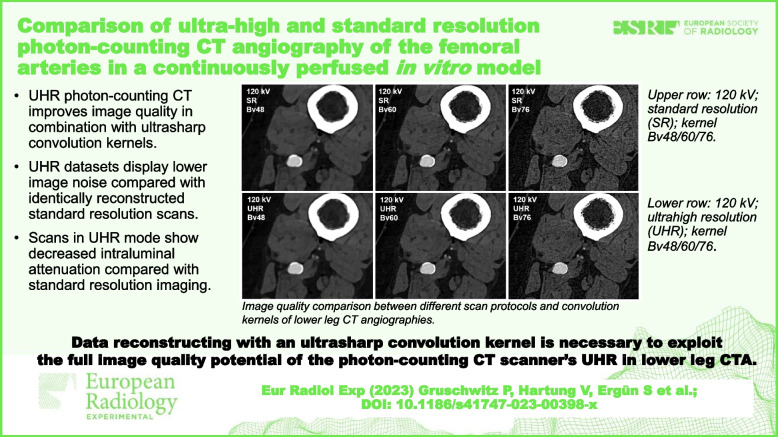

**Supplementary Information:**

The online version contains supplementary material available at 10.1186/s41747-023-00398-x.

## Background

Over the past decade, conventional energy-integrating detector CT has been advanced to its presently conceivable physical limits in terms of dose efficiency and spatial resolution [1]. Particularly with respect to pixel size, constructional restrictions essentially prevent further improvement [2]. Since incident photons are first converted into light before being measured by the diode as a detector signal, opaque septa are necessary to prevent crosstalk between the sensors. Further reduction in pixel size would lead to an increase in dead space, severely limiting dose efficiency [3]. To increase the spatial resolution nevertheless, most conventional scanners employ an aperture-narrowing comb or grid filter positioned in front of the detector and thus behind the patient for ultrahigh resolution (UHR) imaging. In consequence, photons proportional to the incoming x-ray beam are absorbed without contributing to the actual image despite passing through the patient and thus increasing the patient dose [4, 5]. Even a recently released CT scanner equipped with a 0.25 × 160-row detector and detector-sided UHR filter could not overcome the dose disadvantage of UHR imaging entirely [6].

The latest photon-counting detector CT (PCD-CT) technology, however, offers the option of a dose-neutral UHR mode due to the novel detector design without the need for a radiopaque interlayer [7, 8]. Since the conversion of photons into electrical energy occurs in the semiconductor material without the intermediate step of conversion to visible light, septa are no longer required. Hereby, smaller subpixel readout becomes feasible resulting in an effective pixel size of 150 × 176 µm in the isocenter and thereby a relevant increase in spatial resolution [3, 7, 9, 10]. While 4 subpixels are read out together as one unit in standard resolution (SR) mode — a process known as 2 × 2 binning with a resulting z-collimation of 140 rows × 0.4 mm detector pixels — the subpixels are read out separately to acquire images in UHR mode. Due to the resulting increase in data volume, however, the z-collimation is limited to 120 rows × 0.2 mm [7, 11–13]. For cardiac [14–19], pulmonary [20, 21], and musculoskeletal imaging [22–25], several advantages of the UHR scan mode have already been demonstrated. Benefits for imaging of the vascular periphery can be surmised from these data, especially superior delineation of narrow caliber or severely calcified vessels. While this is expected to increase the diagnostic accuracy and confidence, primarily in the lower leg arteries, the actual extent of this effect in clinical routine is yet unclear.

To investigate the UHR mode in direct comparison with the SR mode in photon-counting CT angiography (CTA) of the femoral arteries, this study introduces a cadaveric *in vitro* model with continuous extracorporeal perfusion for objective and subjective image quality assessment.

## Methods

### Human cadaver *in vitro* phantom

This study was performed in accordance with applicable law and the local ethics committee approved the experimental design. To establish the *in vitro* model, 4 cadaveric specimens (2 men, 2 women) were obtained from the local institute of anatomy. Body donors had previously provided informed consent to the use of their cadavers for research and teaching purposes. Of note, 3 of 4 cadavers were used for experiments in a previous study [26]. The femoral vessels of each specimen were continuously perfused with a 13 mg/ml iodine contrast medium solution to achieve a targeted intraluminal attenuation of 400 HU with a standard 120-kV scan in SR mode [27]. Perfusion was established via vascular accesses in the groin and below the knee by means of a pump circuit and examined under flux to simulate a standard pelvic-leg CTA. Details of the experimental setup and the preparation of the cadaveric specimens can be found elsewhere [28].

### Scan protocol and image reconstruction

Upper leg runoff CTA were performed using a clinical PCD-CT (Naeotom Alpha; Siemens Healthcare GmbH, Forchheim, Germany). CT scans were acquired with a consistent radiation dose of 5 mGy and a tube voltage of 120 kV, resulting in an effective tube current of 63 mAs. Both SR (z-collimation 144 × 0.4 mm) and UHR mode (120 × 0.2 mm) were utilized without repositioning of specimens. The pitch factor was set to 0.9 for UHR and 0.4 for SR examinations to match the scan duration between acquisition modes. Of note, the helical pitch is a dimensionless factor depending on the collimation width, which is 57.6 mm for SR *versus* 24 mm for UHR mode. Rotation time was set to 0.5 s.

For each leg runoff, individual polyenergetic reconstructions were prepared with a small field of view measuring 150 mm and a slice thickness/increment of 1.0 mm, respectively. Three different vascular convolution kernels were applied to reformat the images, listed in increasing order of sharpness: Body-vascular-(Bv)48 (soft; ρ50 = 5.40 lp/cm), Bv60 (sharp; ρ50 = 8.79 lp/cm), and Bv76 (ultrasharp; ρ50 = 16.47 lp/cm). A 512^2^ pixel matrix was employed for Bv48 and Bv60, while the matrix for the Bv76 contained 768^2^ pixels according to the default scanner settings. In each case, a 4th-generation iterative reconstruction algorithm at strength level 3 was employed (QIR, Siemens). Since the lowest energy threshold was set to 20 keV by default, images only incorporate x-ray quanta above said threshold in order to eliminate electronic noise.

### Image quality analysis

Four arterial levels (proximal, middle, distal superficial femoral artery, and popliteal artery) were evaluated. Intraluminal attenuation, as well as the attenuation of the surrounding muscle and subcutaneous fat tissue, were measured using standardized regions of interest with a size of 30 mm^2^. Standard deviation of signal attenuation within the unenhancing lipid tissue was deemed representative of image noise. A radiologist with 5 years of experience in CTA imaging performed all measurements using clinical PACS software (Merlin; Phönix-PACS, Freiburg, Germany).

Signal-to-noise ratios (SNR) and contrast-to-noise ratios (CNR) were calculated as $SNR = \frac{HU artery}{SD fat}$ and $CNR = \frac{(HU artery-HU muscle)}{SD fat}$. Image stacks were compared using an in-house programmed forced-choice pairwise comparison tool. Axial-oriented image slices of both legs at the mid-level of the superficial femoral artery were presented, resulting in 48 images and 120 one-on-one comparisons. Six radiologists, including three residents with 1 to 5 years and three board-certified radiologists with 7 to 8 years of clinical training, independently evaluated the luminal assessability and delineation of calcifications using a certified diagnostic monitor (RadiForce RX660; EIZO, Hakusan, Japan) while blinded to any protocol-related information. Data were processed in a Jupyter Notebook environment with Python version 3.8.15 using a Bradley-Terry Model [29] to allow for hierarchical ranking of the compared subgroups, ranging from 1 (best) to 6 (worst) in terms of subjective image quality.

### Statistics

Data is presented as mean ± standard deviation unless differently specified. Statistical analyses were conducted with specialized software (DATAtab e.U., Graz, Austria). Continuous variables are reported as mean ± standard deviation. Significance was determined at an alpha level of *p* < 0.05. Objective image parameters were evaluated with Bonferroni-corrected *t*-tests. Subjective image quality ratings were compared using Friedman tests, and preference ranks were obtained by fitting a Bradley-Terry Model for the entire dataset and for each rater individually [29]. Bump chart diagrams and Boxplots were generated for visualization purposes. Inter-reader agreement was measured using Kendall’s concordance coefficient (*W*).

## Results

**Fig. 1:**
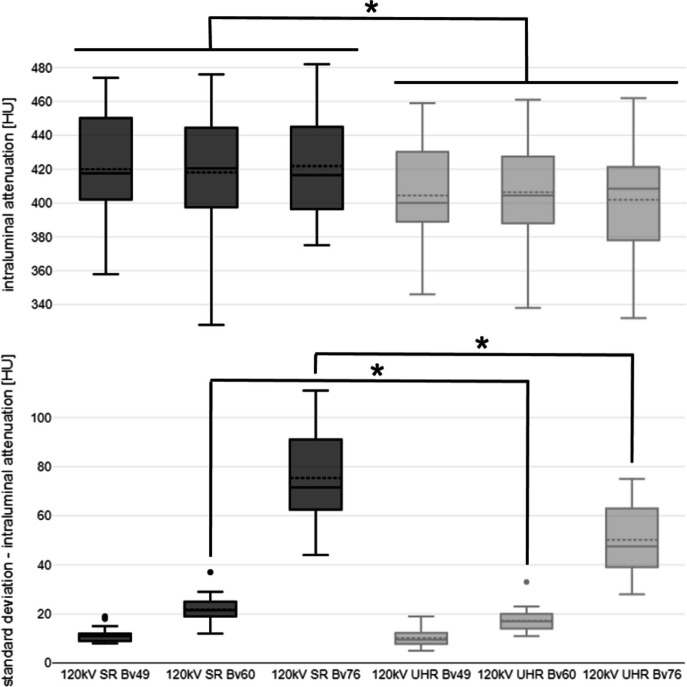
Boxplot illustrating intraluminal attenuation and image noise differences between scan protocols and convolution kernels. *SR* Standard resolution, *UHR* Ultrahigh resolution; asterisk indicates significance level of *p* < 0.001

**Fig. 2:**
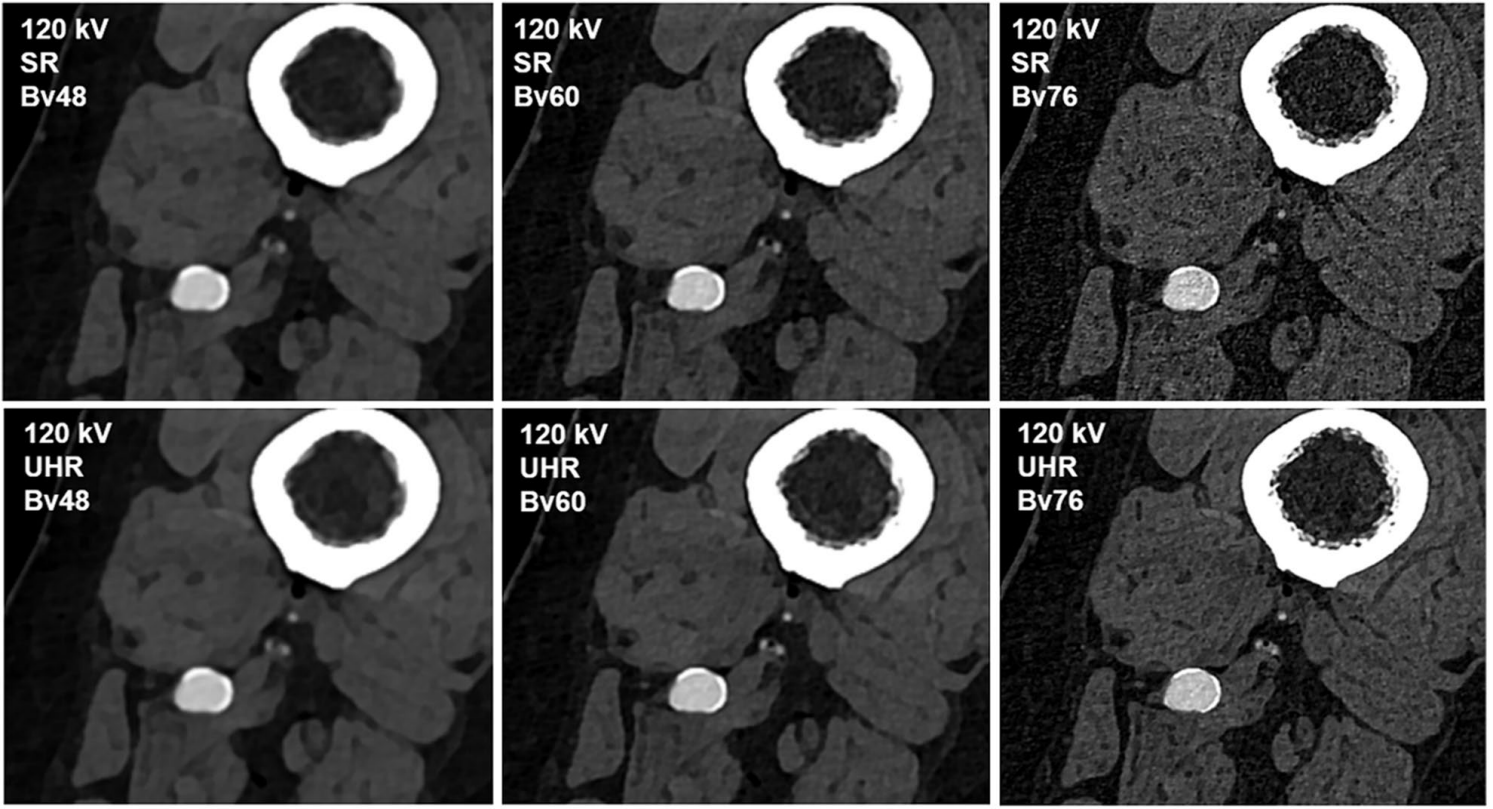
Image quality comparison between different scan protocols and convolution kernels. *Upper row*: 120 kV; standard resolution (*SR*) mode; Bv48/60/76. *Lower row*: 120 kV; ultrahigh resolution (*UHR*) mode; Bv48/60/76

Intraluminal attenuation remained constant between scan modes regardless of the reconstruction kernel used (*p* > 0.999). However, the intraluminal attenuation of scans performed in UHR mode was lower than in the SR mode despite identical tube voltage settings (*e.g.*, Bv60: 406.4 ± 25.1 HU *versus* 418.1 ± 30.1 HU; *p* < 0.001). Image noise increased with sharper reconstruction kernels in all comparisons (*e.g.*, concerning intraluminal measurement, SR: Bv48 11.2 ± 2.1 HU *versus* Bv76 75.4 ± 16.2 HU). However, the image noise associated with sharp and ultrasharp reconstructions (Bv60/76) was significantly lower when using the UHR instead of the SR scan mode for image acquisition (*e.g.*, for intraluminal measurement, UHR: Bv76 50.2 ± 11.7 HU *versus* SR: 75.4 ± 16.2 HU; *p* < 0.001). Intraluminal signal attenuation and standard deviation thereof are summarized in Fig. 1, while a comprehensive display is provided in Table 1. Figure 2 contains representative axial image slices acquired in UHR and SR mode and reconstructed with the three different convolution kernels.

**Table 1:** Objective image quality assessment

**Scan Mode/Kernel**		**Att**_**lum**_ *[HU]*	**SD Att**_**lum**_ *[HU]*	**SD Att**_**musc**_ *[HU]*	**SD Att**_**fat**_ *[HU]*	**SNR**	**CNR**
**SR**	Bv48	419.9 ± 25.6	11.2 ± 2.1	11.1 ± 1.8	11.1 ± 1.1	38.3 ± 4.7	33.7 ± 4.4
	Bv60	418.1 ± 30.1	21.8 ± 3.6	23.5 ± 12.7	21.8 ± 5.2	20.9 ± 5.3	18.4 ± 4.8
	Bv76	421.9 ± 27.9	75.4 ± 16.2	73.9 ± 12.7	69.2 ± 15.9	6.6 ± 1.7	5.8 ± 1.5
**UHR**	Bv48	404.5 ± 24.6	10.1 ± 2.4	10.7 ± 1.4	10.8 ± 1.1	38.0 ± 4.8	33.4 ± 4.4
	Bv60	406.4 ± 25.1	17.2 ± 3.4	19.5 ± 3.8	17.2 ± 4.2	25.9 ± 6.4	22.7 ± 5.8
	Bv76	401.9 ± 26.1	50.2 ± 11.7	49.9 ± 8.9	45.6 ± 11.6	9.7 ± 2.6	8.5 ± 2.3

*Att*_*fat*_ Attenuation of fat tissue, *Att*_*lum*_ Intraluminal attenuation, *Att*_*musc*_ Attenuation of muscle tissue, *CNR* Contrast-to-noise-ratio, *HU* Hounsfield units, *SD* Standard deviation, *SNR* Signal-to-noise-ratio, *SR* Standard resolution, *UHR* Ultrahigh resolution

**Fig. 3:**
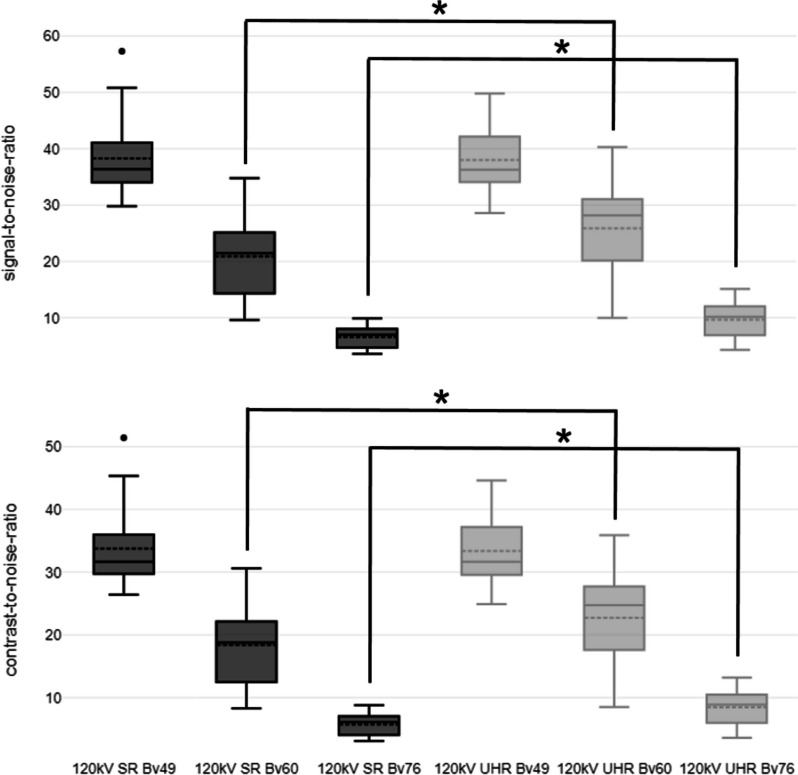
Boxplot illustrating signal-to-noise and contrast-to-noise differences between scan protocols and convolution kernels. *SR* Standard resolution, *UHR* Ultrahigh resolution; asterisk indicates significance level of *p* < 0.001

CNR in SR and UHR mode did not significantly differ when the soft convolution kernel Bv48 was used for image reconstruction (UHR: 33.4 ± 4.4 *versus* SR: 33.7 ± 4.4; *p* = 0.722). However, CNR was significantly higher for UHR acquisitions when the sharp kernel Bv60 (UHR: 22.7 ± 5.8 *versus* SR: 18.4 ± 4.8; *p* < 0.001) or the ultrasharp kernel Bv76 were employed (UHR: 8.5 ± 2.3 *versus* SR: 5.8 ± 1.5; *p* < 0.001). A similar tendency was observed for SNR, which was significantly higher for reconstructions of UHR data with the sharp and ultrasharp kernels (*p* < 0.001, each), while no significant difference was observed for reconstructions with the soft kernel (*p* = 0.799). CNR and SNR results are summarized in Table 1 and visualized in Fig. 3.

**Fig. 4:**
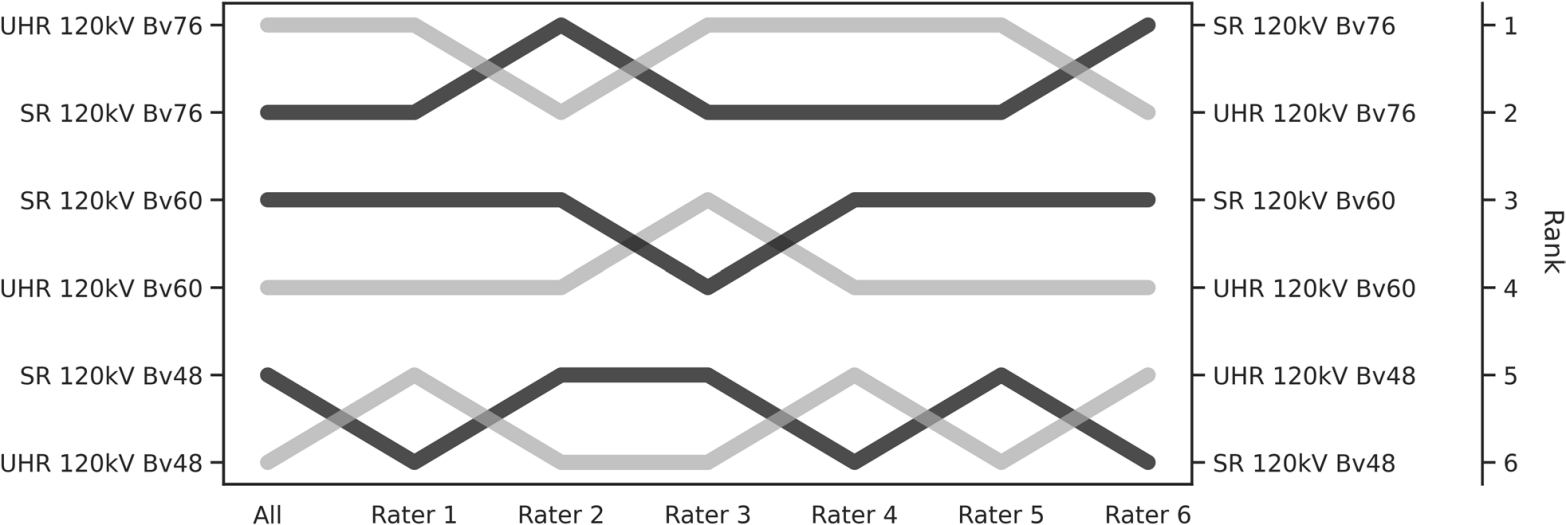
Bump chart of the subjective image quality ratings. A bump chart shows the results of subjective image quality evaluation in the form of ranks based on the pairwise forced-choice assessment for each combination of scan mode and reconstruction kernel. Ratings of radiologists are given individually (*Rater 1 to 6*) and in pooled fashion (*All*)

Regardless of scan mode, sharp reconstructions were deemed superior to images generated with soft kernels. The UHR images reconstructed with the ultrasharp kernel Bv76 received the highest observer ratings, followed by SR images reconstructed with the same kernel. While a measurable improvement in CNR/SNR was recorded for UHR mode acquisitions when the sharp kernel Bv60 was used for image reconstruction, the raters generally preferred the SR mode. When the soft reconstruction kernel Bv48 was used, no preference was observed between SR and UHR mode. Inter-reader agreement was high (*W* = 0.935) with individual rankings visualized in form of a bump chart in Fig. 4. Ranking differences were significant in all cases (*p* < 0.001).

## Discussion

This experimental study on four continuously perfused human cadaver models evaluated the influence of standard and ultrahigh-resolution scan mode on image quality in photon-counting CT angiographies of the femoral arteries dependent on the reconstruction kernel used under standardized conditions. Our results indicate the superiority of a combination of ultrahigh-resolution scan mode and ultrasharp image reconstruction based on quantitative assessment and multiobserver analysis in a pairwise forced-choice setup.

A recent investigation was able to demonstrate the dose reduction and image quality optimization potential of PCD-CT in SR mode compared to energy-integrating detector CT using similar scanner settings [28]. Of note, the authors stated that the SR scan mode was deliberately used instead to achieve maximal comparability. While most radiation dose-defining settings (*e.g.*, tube voltage, effective mAs) were chosen in accordance with this aforementioned study, we consciously selected a single radiation dose level of 5 mGy for the present work, which was deemed appropriate for clinical use and is below the applicable national guideline value. In contrast to prior studies on CTA of the vascular periphery, this research, to the authors’ best knowledge, is the first to provide direct side-by-side comparisons between UHR and SR scan mode in a fully standardized *in vitro* environment.

Notably, the choice of reconstruction kernel had no significant influence on intraluminal attenuation. However, somewhat surprisingly, the mean intraluminal attenuation at the same tube voltage (120 kV) was recorded to be significantly lower in UHR mode than in SR mode. Potential dilution effects during the cadaver perfusion, *e.g.*, due to extravasation of contrast medium, could be ruled out in a supplementary experiment, in which cylindrical sample tubes holding different concentrations of iodine solution were scanned with the same acquisition settings (Supplemental Fig. S1). For this reason, a physical cause of the observed difference is conceivable, but more studies are needed to evaluate this effect further.

Consistent with the literature on cardiac PCD-CT, overall image noise increased with higher spatial frequency [14, 16, 18]. However, in accordance with a previous study by Leng et al. [10], this increase in noise could be significantly reduced by scanning in UHR mode. Even though SNR and CNR were numerically lowest for reconstructions with the ultrasharp Bv76 kernel, observers preferred this post-processing setting, especially if the reconstructed data was acquired in UHR mode. This finding is generally in line with recent musculoskeletal imaging studies, which also postulated the best subjective results for the sharpest investigated kernels [30, 31]. Within each scan mode, images reconstructed with sharper kernels were subjectively rated better in the forced-choice comparison setup. This tendency may be explained by a more detailed visualization of the vessel wall and thus superior delineation of calcified and noncalcified plaques. Also allowing for reduced image noise, image acquisition in UHR mode holds potential to increase the diagnostic accuracy of CTA, particularly in narrow-caliber and/or circularly calcified vessels, *e.g.*, in advanced peripheral arterial disease [32, 33]. In that regard, promising results have already been demonstrated in cardiac CT [14–17] and phantom studies [19, 34, 35]. While SR mode was preferred over UHR mode for reconstructions with the sharp Bv60 kernel, we attribute this observation to the image impression more closely resembling the familiar output of EID-CT scanners used in our department. Although CTA is usually focused on the depiction of contrasted vessels, it must be mentioned that the assessability of the adjacent soft tissues is reduced with increasing spatial frequency of the convolution kernel. Therefore, reconstructions with more than one kernel may be necessary for a comprehensive evaluation of the vascular runoff and surrounding soft tissue. In that regard, a sharp kernel (*e.g.*, Bv64) may serve as a compromise [16]. Since the pixel matrix also has considerable influence on the image noise level [36], the vendor-specific default settings were used in the present study.

The primary reason for the recorded noise reduction in UHR mode lies presumably in the “small pixel effect,” a result of the smaller detector pixel size and therefore downscaled focal spot in fan direction [37]. On the reconstruction side, in order to realize a certain degree of sharpness, the convolution kernel dampens the enhancement of high frequencies, resulting in a reduced noise increase. On one side, this phenomenon can be exploited to produce scans with a lower radiation dose while maintaining the same noise level compared to SR data. On the other side, the small pixel effect allows for noise reduction with the same radiation dose [7, 38]. Due to the predominantly elderly population receiving pelvic-leg CTA, better image quality may be preferred over dose reduction in these patients. Presumably, more precise radiological assessment can lead to a reduction of potentially unnecessary invasive examinations in clinical routine. In this context, virtual low keV reconstructions facilitating contrast agent reduction are also of high interest and warrant further studies.

For the present investigation, image reconstructions were deliberately performed with a slice thickness of 1 mm. In principle, a lower slice thickness can be selected, but the diagnostic gain is questionable due to increasing image noise. In support, a patient study by Milos et al. [39] comparing PCD-CT of the lung in UHR mode with different convolution kernels showed that image noise increases significantly with decreasing slice thickness. While this study did not provide a control group with scans in SR mode, it can be assumed that the observed indirect relationship between image noise and slice thickness is at least equally pronounced in SR mode. With regard to the present work, the relative noise reduction in UHR mode may be even more pronounced at lower reconstructed slice thicknesses, benefitting especially the diagnostic evaluation of the lower leg runoff.

UHR scan mode should next be evaluated with respect to diagnostic accuracy, especially for small vessel diameters (*e.g.*, in the lower leg) and in the presence of high-grade calcifications. Based on a recent cardiac CT study by Rajendran et al. [40], the postulated advantages of UHR examinations may also be translatable to arteriosclerotic alterations in peripheral vessels. While additional comparative studies are mandated, first publications recommend the use of UHR scan mode in principle for all PCD-CT examinations [41]. In the context of smaller scan volumes, *e.g.*, in cardiac or musculoskeletal imaging, this statement may be valid, but the considerably increased raw data file size of UHR compared to SR acquisitions is certainly relevant for larger scan volumes at this time [7]. Of note, file size has become a pertinent cost factor both in terms of computation time and energy consumption, which is to be seen critically considering climate change and the overall need for “greener” medicine [42].

In addition to being a single-center, single-scanner investigation, some limitations of this study must be mentioned. First, due to the experimental and laborious study design, only 8 lower extremities of 4 body donors were examined. With the current design of the perfusion model, only large-lumen femoral arteries could be examined, however, the largest diagnostic benefit of UHR scan mode can certainly be expected for the narrow caliber arteries of the lower leg. Second, to increase comparability and provide a clinically applicable imaging setup, we employed a single tube voltage of 120 kV and constant CT dose index of 5.0 mGy, while reconstructing all data with a slice thickness/increment of 1.0 mm. To maintain realistic scan durations among acquisition modes, the spiral pitch factor had to be increased for UHR studies. Third, the implications of using lower tube voltages were not investigated. Similarly, spectral post-processing applications, such as virtual monoenergetic imaging, were not in the scope of the present study. Further studies are necessary in that regard.

To conclude, in photon-counting CT angiographies of the femoral arteries, reconstructing data with an ultrasharp convolution kernel is necessary to exploit the full image quality potential of the scanner’s ultrahigh-resolution scan mode.

### Supplementary Information

**Additional file 1: Supplemental Fig. S1.** Sample tubes with varying iodine concentrations scanned in standard and ultrahigh resolution mode. Iodine concentration left to right: 100 mg/mL; 58 mg/mL; 41 mg/mL; 32 mg/mL; 22 mg/mL; 17 mg/mL; 11 mg/mL. A) Standard resolution (*SR*) mode; 120 kV; Bv60; 5 mGy. B) Ultrahigh resolution (*UHR*) mode; 120 kV; Bv60; 5 mGy. *SD* Standard deviation.

## Data Availability

The datasets generated and/or analyzed during the current study are not publicly available but are available from the corresponding author on reasonable request. Due to the nature of this research, participants of this study did not agree to their data to be shared in a public repository.

## Abbreviations

Bv: Body vascular (convolution kernel)
CNR: Contrast-to-noise ratio
CTA: Computed tomography angiography
PCD-CT: Photon-counting detector computed tomography
SNR: Signal-to-noise ratio
SR: Standard resolution
UHR: Ultrahigh resolution
