# Supplementary material for: Comparison of ultrahigh and standard resolution photon-counting CT angiography of the femoral arteries in a continuously perfused in vitro model
Source: Eur Radiol Exp. 2023 Dec 18;7:83. doi: 10.1186/s41747-023-00398-x (PMC10728414; doi:10.1186/s41747-023-00398-x)
Supplement: Supplementary file 1 — Additional file 1: Supplemental Fig. S1. Sample tubes with varying iodine concentrations scanned in standard and ultrahigh resolution mode. Iodine concentration left to right: 100 mg/mL; 58 mg/mL; 41 mg/mL; 32 mg/mL; 22 mg/mL; 17 mg/mL; 11 mg/mL. A) Standard resolution (SR) mode; 120 kV; Bv60; 5 mGy. B) Ultrahigh resolution (UHR) mode; 120 kV; Bv60; 5 mGy. SD Standard deviation. [file 41747_2023_398_MOESM1_ESM.docx]

**Comparison of ultrahigh and standard resolution photon-counting CT angiography of the femoral arteries in a continuously perfused *in vitro* model**

**ELECTRONIC SUPPLEMENTARY MATERIAL**

**
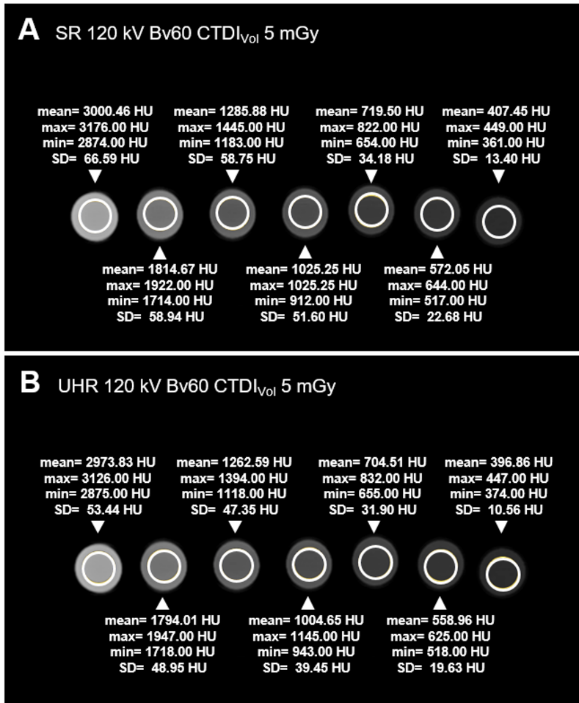
**

**Supplemental Fig. S1.** Sample tubes with varying iodine concentrations scanned in standard and ultrahigh resolution mode. Iodine concentration left to right: 100 mg/ml; 58 mg/mL; 41 mg/mL; 32 mg/mL; 22 mg/mL; 17 mg/ml; 11 mg/mL. **A)** Standard resolution (*SR*) mode; 120 kV; Bv60; 5 mGy. **B)** Ultrahigh resolution (*UHR*) mode; 120 kV; Bv60; 5 mGy. *SD* Standard deviation**.**
